# Profound Effects of Population Density on Fitness-Related Traits in an Invasive Freshwater Snail

**DOI:** 10.1371/journal.pone.0080067

**Published:** 2013-11-21

**Authors:** Nicholas Zachar, Maurine Neiman

**Affiliations:** Department of Biology, University of Iowa, Iowa City, Iowa, United States of America; Tel Aviv University, Israel

## Abstract

Population density can profoundly influence fitness-related traits and population dynamics, and density dependence plays a key role in many prominent ecological and evolutionary hypotheses. Here, we evaluated how individual-level changes in population density affect growth rate and embryo production early in reproductive maturity in two different asexual lineages of *Potamopyrgus antipodarum*, a New Zealand freshwater snail that is an important model system for ecotoxicology and the evolution of sexual reproduction as well as a potentially destructive worldwide invader. We showed that population density had a major influence on individual growth rate and early-maturity embryo production, effects that were often apparent even when comparing treatments that differed in population density by only one individual. While individual growth rate generally decreased as population density increased, we detected a hump-shaped relationship between embryo production and density, with females from intermediate-density treatments producing the most embryos and females from low- and high-density treatments producing the fewest embryos. The two lineages responded similarly to the treatments, indicating that these effects of population density might apply more broadly across *P. antipodarum*. These results indicate that there are profound and complex relationships between population density, growth rate, and early-maturity embryo production in at least two lineages of this important model system, with potential implications for the study of invasive populations, research on the maintenance of sex, and approaches used in ecotoxicology.

## Introduction

*Potamopyrgus antipodarum*, a freshwater snail native to New Zealand, has achieved prominence as the focus of a large body of research aimed at understanding the maintenance of sexual reproduction (*e.g.*, [1]–[3]), as a destructive invasive species [4], and as an emerging ecotoxicology model system (*e.g.*, [5]–[7]). These studies typically use traits like reproductive output and individual growth rate to, for example, estimate the relative fitness of sexual vs. asexual individuals (*e.g.*, [8]), measure responses to environmental pollutants (*e.g.*, [9]), and evaluate sensitivity of invasive populations to nutrient limitation (*e.g.*, [10]).

While these studies have certainly provided important advances, the growing body of evidence that *P. antipodarum* is very sensitive to environmental variables like food availability [11], population density [11]–[13], temperature [7], [14]–[15], and dietary nutrient content [10], [16] means the careful use of *P. antipodarum* as a model system requires a thorough understanding of how fitness-related traits are influenced by environmental conditions [13]. A number of recent studies have made a great deal of progress in this direction [7], [10]–[11], [15]–[17], but critical questions remain. For example, despite the complex relationship between *P. antipodarum* reproduction and population density [11]–[13] and the clear sensitivity of *P. antipodarum* to food availability [11], no study has simultaneously controlled per-capita food and measured the response of fitness-related traits across a series of population densities. As such, we still do not have a clear understanding of the nature of the relationship between population density per se and fitness-related traits in *P. antipodarum* or know whether the positive relationships between population density and reproductive output observed in Neiman et al. (2013) [11] and Sieratowicz et al. (2013) [13] extends to the much higher population densities often used in ecotoxicology studies (*e.g.*, [9]) and observed in invasive populations (*e.g.*, [18]–[19]). Furthermore, because most studies of the relationships between fitness-related traits and environmental variables in *P. antipodarum* do not include individuals from multiple genetically distinct asexual lineages (*e.g.*, [10]–[13]), the extent to which the conclusions of these studies apply across this notably diverse species [20], [21] remains unclear.

Here, our goal was to provide an in-depth characterization of the relationship between population density, growth rate, and reproductive output in *P. antipodarum*. Our main points of departure from similar studies conducted by Neiman et al. (2013) [11] and Sieratowicz et al. (2013) [13] are 1) the combined use of a wide range of population densities and precisely controlled food administration, 2) individual-level variation in population density, and 3) the inclusion of two genetically distinct lineages. Because growth and reproduction in *P. antipodarum* is very sensitive to food availability [11], parsing out direct effects of population density requires that food availability does not vary across density treatments. Furthermore, changes in population density at the scale of one or a few individuals can influence growth and reproduction in *P. antipodarum* [11], [22], meaning that the use of individual-level population density treatment intervals are required to characterize the relationship between density and fitness in *P. antipodarum*. Finally, the use of two genetically distinct lineages of *P. antipodarum* enables us to both evaluate whether there is genetic variation for response to population density and determine whether our results might be more broadly generalizable. We were able to address all of these points, finding that in each of two genetically distinct *P. antipodarum* lineages, growth rate decreases with increased population density and the relationship between reproductive output and population density is hump-shaped.

## Materials and Methods

We established our experiment with 207 arbitrarily selected juvenile *P. antipodarum* (<2 mm in shell length (*e.g.*, [17]) from each of two different asexual triploid lineages. One lineage was descended from a single asexual female sampled in January 2009 from Lake Waikaremoana (North Island, New Zealand, “Waikaremoana”), and the other lineage was descended from a single asexual female sampled in 2008 from a stream entering Lake Ontario, New York (“Ontario”). *Potamopyrgus antipodarum* is not an endangered or protected species, and necessary permits were granted by the New Zealand Department of Conservation, the New York State Department of Environmental Conservation, and the Iowa Department of Natural Resources.

Within each lineage, we arbitrarily assigned each individual to one of ten population density treatments, which consisted of 2, 3, 4, 5, 6, 7, 8, 9, 10, or 15 individuals housed in 1-liter polypropylene cups filled with ∼300 mL of carbon-filtered water (∼256–1923 snails/m^2^). The population densities used in our density treatments are within the range of population densities observed in natural and invasive populations of *P. antipodarum*, which themselves vary widely in density (*e.g.*, 200–25,000 snails/m^2^ [18]; 1700–49,000 snails/m^2^ [19]; 22–299,000 snails/m^2^ [23]). Each population density level was replicated three times per lineage, for a total of 60 replicated sample populations. Each replicate received a solution containing 0.023 mg dried *Spirulina* per snail, a standard diet for laboratory *P. antipodarum* (*e.g.*, [11]), three times per week. Water was changed weekly. We checked each cup for dead snails once a week, replacing dead individuals with a similarly sized individual from the same lineage in order to maintain population density within each replicate. Fifteen of the 414 snails (3.6%; four Waikaremoana and 11 Ontario) died over the course of the experiment; the fifteen “replacement” individuals were not included in data analyses.

We used nail polish to mark the shell of each individual with a unique color identifier prior to the start of experimental treatments. Each individual was photographed under a dissecting microscope next to a metric ruler at the beginning of the experiment and shell length was quantified using ImageJ software. We followed the same procedure every four weeks in order to measure growth in shell length. Because female *P. antipodarum* reach reproductive maturity at or above ∼3.0 mm in shell length [17], [24], we dissected all individuals within a lineage once the mean lineage shell length within any given population density treatment level exceeded 3.0 mm in order to ensure that we quantified embryo production in these ovoviviparous snails at this early period in reproductive maturity. As an additional means of ensuring that we were counting embryo production at the beginning of reproductive maturity, we also periodically dissected (and then replaced again) the replacement females (described above) to check for the presence of brooded embryos. If one or more of these females contained embryos, we then dissected all of the experimental snails within the lineage in order to quantify embryo production. The Waikaremoana lineage met all criteria for reproductive maturity at day 183 of the experiment (April 16–October 16, 2012), and all Waikaremoana snails were dissected at this time after a final shell length measurement. The Ontario lineage took an additional 30 days to meet reproductive maturity criteria (213 days; April 16–November 15, 2012), upon which we measured and dissected all Ontario snails.

### Statistical analyses

Paired samples t-tests indicated that there were significant differences in shell length between each consecutive set of shell length measurements for each lineage (*p*<0.0001 for all comparisons; data not shown). Because this meant that snails were still experiencing substantial growth at the conclusion of the experiment, we used the total increase in shell length over the course of the experiment for each snail as our primary growth rate response variable. Following Sterner and Elser (2002) [25], we then estimated specific growth rate for each individual snail (“SGR”) as: ln(shell length at end of experiment/shell length at beginning of experiment)/duration of the experiment in days.

Next, we used these SGR values to calculate the mean SGR per replicate population, and then used these SGR replicate means in order to control for non-independence of individuals that were housed together within cups. We then used the Shapiro-Wilk test to evaluate whether the mean SGR data met the normal distribution requirement of parametric statistical analysis. The distribution of these data departed significantly from a normal distribution (Shapiro-Wilk test statistic 0.929, df = 60, *p* = 0.002), so we used a log_10_ transformation to transform the data. The distribution of the log-transformed mean SGR data did not depart significantly from a normal distribution (Shapiro-Wilk test statistic = 0.968, df = 60, *p* = 0.117), so we were able to use these data as the dependent variable in a univariate ANOVA with population density as a fixed factor and lineage as a random factor. We used a post-hoc Tukey's honestly significant difference analysis to identify differences in mean log SGR between particular population density levels.

We took a very similar approach with the embryo count data, first calculating mean replicate embryo number in order to control for the non-independence of snails that were housed together within cups. The distribution of replicate embryo number means did not depart significantly from a normal distribution (Shapiro-Wilk test statistic = 0.970, df = 60, *p* = 0.149), so we were able to use these embryo number replicate means for subsequent analyses. We then used the same univariate ANOVA model structure as described for SGR to determine how the population density treatments and lineage identity affected mean population embryo production. Because positive shell length-fecundity relationships have been reported in female *P. antipodarum* [10], [17], we included final shell length as a covariate in this analysis. Final shell length was not significantly correlated with embryo production, however (*F*_1, 39_ = 0.567, *p* = 0.456), so we then ran another analysis without this covariate but with an otherwise identical model structure. We used a post-hoc Tukey's honestly significant difference analysis to identify differences in mean embryo production between particular population density levels. All of these analyses were conducted with IBM SPSS Statistics v. 21.0.

## Results

**Figure 1:**
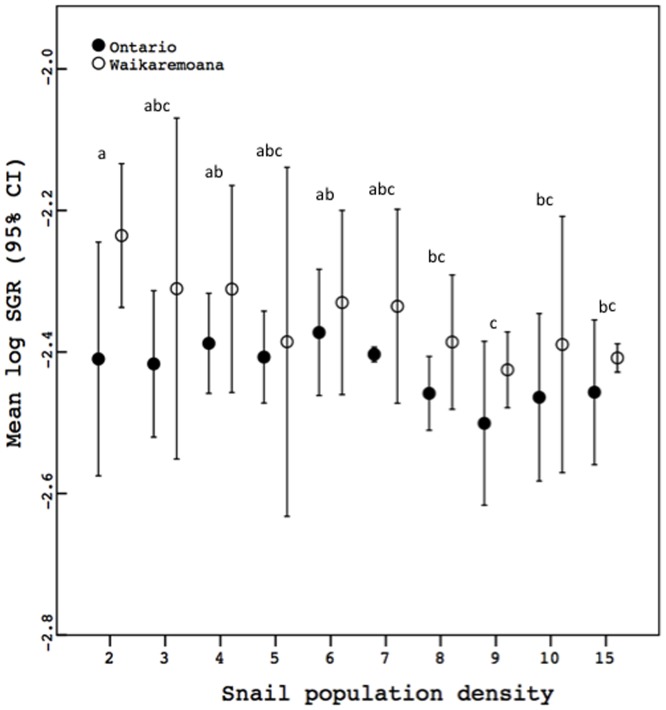
Mean log-transformed SGR per female across the ten population density treatments. Shared letters above error bars denote means (pooled across lineages; Tukey's honestly significant difference) that are not significantly different, while different letters indicate that *p*<0.05 for pairwise comparisons. *p* (a) = />0.309; *p* (b) = />0.171; *p* (c) = />0.051.

Population density significantly affected the SGR of juvenile *P. antipodarum* (Fig. 1, Table 1). Spearman's correlation analyses evaluating the association between population density and SGR indicated that this effect was driven by decreased SGR in higher-density treatments relative to lower-density treatments (Fig. 1; both lineages, Spearman's ρ = −0.466, *p*<0.0001; Ontario: Spearman's ρ = −0.529, *p* = 0.003; Waikaremoana, Spearman's ρ = −0.593, *p* = 0.001). There was also a significant main effect of lineage on SGR, demonstrating the existence of genetic variation for growth rate (also see [16]). There was not a significant density by lineage interaction, indicating that growth rate in the Waikaremoana and Ontario lineages responded similarly to the population density treatments (Fig. 1, Table 1).

**Table 1:** Results of a univariate ANOVA comparing mean replicate SGR (log_10_ transformed) across the ten population density treatments and the two lineages.

Factor	df(error)	MS(error)	*F*	*p*
Population Density	9(9)	0.012(0.003)	4.73	0.015
Lineage	1(9)	0.087(0.003)	33.537	<0.0001
Population Density*Lineage	9(40)	0.003(0.003)	0.976	0.474

**Figure 2:**
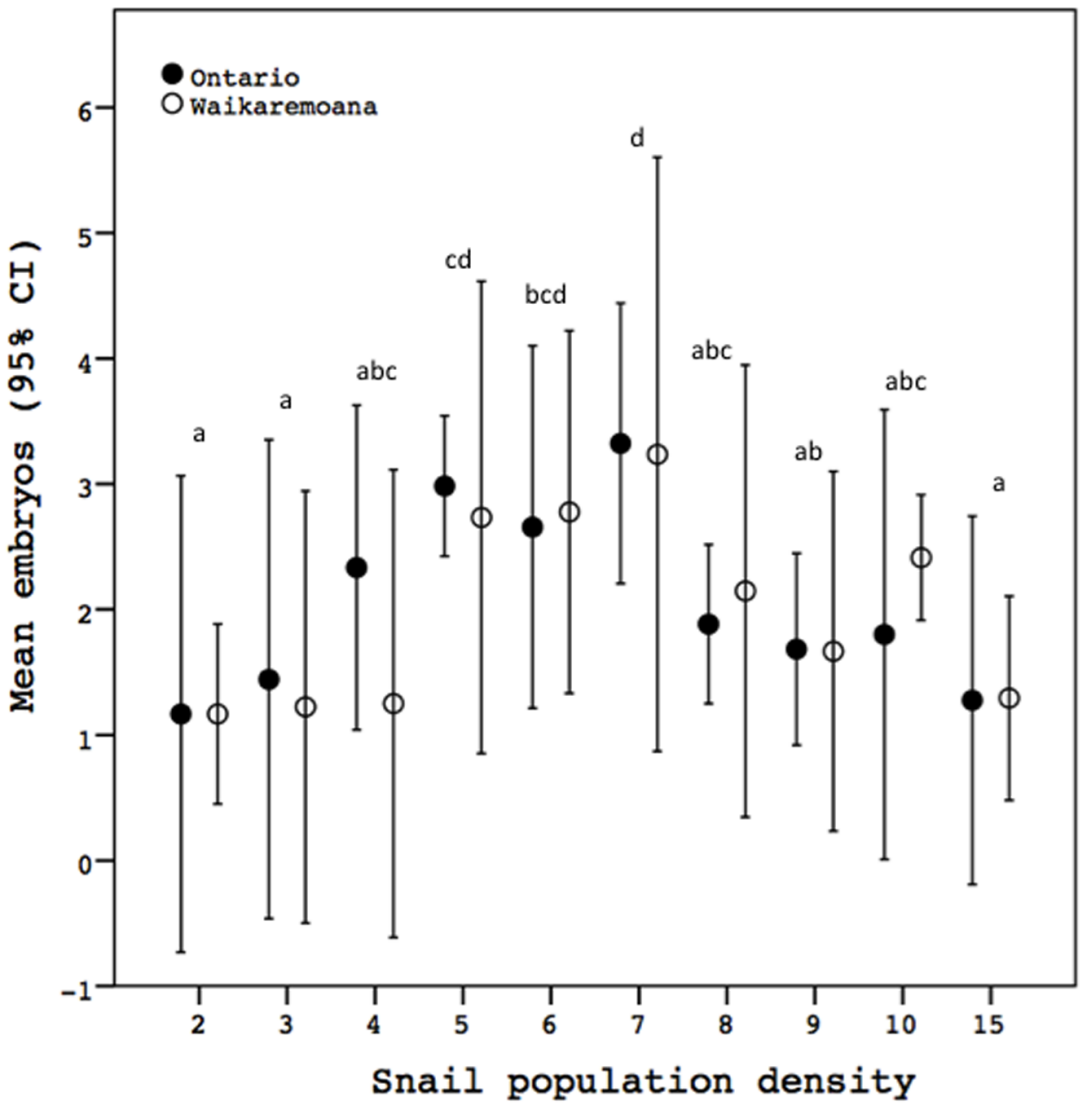
Mean embryo production per female across the ten population density treatments. Shared letters above error bars denote means (pooled across lineages; Tukey's honestly significant difference) that are not significantly different, while different letters indicate that *p*<0.05 for pairwise comparisons. *p* (a) = />0.189; *p* (b) = />0.101; *p* (c) = />0.086; *p* (d) = />0.817.

Population density also had a significant effect on embryo production (Fig. 2, Table 2). Post-hoc Tukey tests revealed that these effects were complex and hump-shaped, with the highest embryo production at intermediate population densities and the lowest embryo production at both high and low population density (Fig. 2). Embryo production was not influenced by lineage or by the interaction between population density and lineage, suggesting that embryo production, like growth rate, responded similarly to the density treatments in the two lineages (Fig. 2, Table 2). The total number of embryos produced by snails in the most productive treatments (mean ∼ three embryos/snail at seven snails/cup) was somewhat low relative to some (*e.g.*, [5], [12], [26]) but not all (*e.g.*, [7], [9], [11]) earlier laboratory-based studies that quantified embryo production in *P. antipodarum*. One possible explanation for the lower embryo production of the snails in our study relative to several other studies is that the *P. antipodarum* in our study had only very recently reached reproductive maturity, though we cannot exclude the possibility that some unknown stressor may have inhibited reproduction.

**Table 2:** Results of a univariate ANOVA comparing mean replicate embryo production across the ten population density treatments and the two lineages.

Factor	df(error)	MS(error)	*F*	*p*
Population Density	9(9)	3.13(0.285)	10.974	0.001
Lineage	1(9)	0.062(0.285)	0.218	0.652
Population Density*Lineage	9(40)	0.285(0.351)	0.813	0.607

## Discussion

We found that specific growth rate of juvenile *P. antipodarum* was markedly decreased at higher population densities and that embryo production by adult female *P. antipodarum* was lowest in the relatively high and relatively low population density treatments and highest at intermediate population densities. There was no difference between the two *P. antipodarum* lineages in how SGR and embryo production responded to the different population density treatments. While direct inferences to *P. antipodarum* as a whole and to natural populations are impossible given that the study included only two laboratory-raised lineages and took place in a laboratory setting, the similarity in response of growth rate and especially embryo output of these different two lineages suggests that our results might be informative with respect to other *P. antipodarum* lineages and/or to natural *P. antipodarum* populations (also see [11]).

### Effect of population density on SGR

Specific growth rate generally decreased as population density increased, with snails in the lowest density treatment (two snails) growing ∼126% more than snails in the highest density treatment (fifteen snails). Such growth rate responses could plausibly translate into fitness differences because *P. antipodarum* females that grow more slowly reach reproductive maturity later than females that grow more rapidly [10], and because fecundity and the rate of achieving reproductive maturity are both positively correlated with female *P. antipodarum* shell length [10], [17], [24].

A negative effect of population density on growth rate in *P. antipodarum* was also reported by Cope and Winterbourn (2004) [22]. Our study, however, is the first to demonstrate that these negative effects of population density on growth rate in *P. antipodarum* occur even when per-capita food is held constant across density treatments. Our study also departed from that of Cope and Winterbourn [22] by starting with juvenile snails that were <2 mm in shell length, much smaller (and likely, younger) than the <3.5 mm snails that they used. Our study thus provides a more complete picture of the influence of population density on growth of *P. antipodarum* from juvenile to adult. Similar negative effects of population density on individual growth rate have been reported from other snail species (*e.g.*, [22], [27]–[29]), though these studies have generally not addressed whether negative density-dependence is also observed in the absence of food limitation.

### Effect of population density on embryo production

Changes in population density exerted a complex, non-linear effect on reproductive output in *P. antipodarum*. The most striking example is provided by comparisons between the seven snail (intermediate density), two snail (lowest density), and fifteen snail (highest density) populations: females from the seven-snail treatment produced ∼281% more embryos than females in the two-snail populations and ∼255% more embryos than females in the fifteen-snail populations. These results are consistent with those of Neiman et al. (2013) [11], who found that embryo production increased by about twofold in populations consisting of six adult female *P. antipodarum* vs. populations with three adult female *P. antipodarum*. Profound effects of population density on embryo production were even observable between treatments differing in density by only one snail, exemplified by the ∼162% higher embryo production of individuals in the seven-snail treatment relative to individuals in the eight-snail treatment. While another recent study of the influence of population density on reproduction in *P. antipodarum* also showed that reproductive output increased at intermediate densities [13], our study is the first to show that this phenomenon occurs in the absence of systematic changes in food availability across density treatments.

Several other studies of effects of population density on reproductive output in other snails have reported negative effects of higher population density on reproduction (*e.g.*, [27], [30]). However, none of these studies have both carefully controlled food and used a range of population density treatments. Because reproductive output in many gastropods is known to be very sensitive to food limitation (*e.g.*, [29], [31]), it is impossible to rule out the potential for food limitation (rather than population density per se) as the source of these effects. It is also possible that more complex relationships between population density and reproductive output will be revealed for at least some of these species if a wider range of density treatments are explored.

### Connections to natural populations, invasive populations, and the use of Potamopyrgus antipodarum for laboratory studies

Our results provide the first in-depth characterization of the relationships between population density, individual growth rate, and early-maturity embryo production in *P. antipodarum*. This information is of direct relevance to researchers who use *P. antipodarum* for applied (e.g., invasion biology, ecotoxicology) and basic (e.g., maintenance of sex) research.

Because variation in life-history traits like growth rate and fecundity can affect population dynamics [32]–[34], it is possible that the density dependence we have observed can influence the dynamics of native and invasive *P. antipodarum* populations. For example, the connections between population density and fitness-related traits that we observed might help to explain previously observed rapid fluctuations in the densities of invasive populations of *P. antipodarum* [18]–[19], [23]. Future studies addressing whether the results we observed are replicated in other *P. antipodarum* lineages and in more natural settings, whether native and invasive *P. antipodarum* respond similarly to changes in population density, and connecting the individual-level effects of population density on fitness-related traits to population dynamics are the next steps in addressing this possibility.

The results of our study are also of direct relevance to the many researchers who use quantification of reproductive output in *P. antipodarum* to study the biological consequences of pollutants and contaminants (*e.g.*, [7], [9], [35]). Our data suggest that future ecotoxicology studies using *P. antipodarum* will need to carefully consider potential influences of population density as experiments are designed and data are interpreted.

Finally, the prominence of *P. antipodarum* as a model system for the study of the maintenance and distribution of sexual reproduction and its extreme sensitivity to changes in population density suggest that future studies might focus on whether the responses of sexual vs. asexual *P. antipodarum* to alterations in population density are different. For example, a more severe reduction of growth rate and/or embryo production for asexual vs. sexual *P. antipodarum* experiencing relatively high population density could help to offset the costs of sexual reproduction. Determining whether *P. antipodarum* show similar sensitivity to changes in population density under field conditions would also present an important step forward in characterizing the biology of this fascinating species.
